# Distribution and molecular characteristics of rickettsiae found in ticks across Central Mongolia

**DOI:** 10.1186/s13071-017-1981-3

**Published:** 2017-02-02

**Authors:** Bazartseren Boldbaatar, Rui-Ruo Jiang, Michael E. von Fricken, Sukhbaatar Lkhagvatseren, Pagbajab Nymadawa, Bekhochir Baigalmaa, Ya-Wei Wang, Benjamin D. Anderson, Jia-Fu Jiang, Gregory C. Gray

**Affiliations:** 1 Institute of Veterinary Medicine, Ulaanbaatar, Mongolia; 2 State Key Laboratory of Pathogen and Biosecurity, Beijing Institute of Microbiology and Epidemiology, Beijing, China; 3 Division of Infectious Disease, Duke Global Health Institute, Duke University, Durham, NC USA; 4 Department of Global and Community Health, George Mason University, Fairfax, VA USA; 5 Mongolian Academy of Medical Sciences, Ulaanbaatar, Mongolia; 6 National Center for Zoonotic Diseases, Ulaanbaatar, Mongolia

**Keywords:** *Rickettsia*, Mongolia, *Ixodes persulcatus*

## Abstract

**Background:**

Little is known regarding tick-borne diseases in Mongolia, despite having 26% of the population still living nomadic pastoral lifestyles. A total of 1497 adult unfed ticks: 261 *Ixodes persulcatus*, 795 *Dermacentor nuttalli*, and 441 *Hyalomma asiaticum*, were collected from three ecologically distinct regions in Central Mongolia. Tick pools (*n* = 299) containing ~5 ticks each, were tested for *Rickettsia* and Tick-borne encephalitis virus (TBEV) using nested polymerase chain reaction, reverse transcription-PCR, and quantitative real-time RT-PCR.

**Results:**

Assays yielded pooled prevalence of 92.5% (49/53) and 1.9% (1/53) for pooled *I. persulcatus* testing positive for “*Candidatus* Rickettsia tarasevichiae” and TBEV, respectively, while *Rickettsia raoultii* was found in 72.8% (115/158) of pooled *D. nuttalli* samples. When calculating a maximum likelihood estimation, an estimated 46.6% (95% CI: 35.2–63.6%) of *I. persulcatus* ticks in the pooled sample were infected with “*Candidatus* R. tarasevichiae”.

**Conclusions:**

Both “*Candidatus* R. tarasevichiae” and *R. raoultii* are recognized as emerging tick-borne pathogens, with this being one of the first reports of “*Candidatus* R. tarasevichiae” in Mongolia. Given that “*Candidatus* R. tarasevichiae” shares the same vector (*I. persulcatus*) as TBEV, and infections may present with similar symptoms, Mongolian physicians treating suspected cases of TBEV should include “*Candidatus* R. tarasevichiae” infection in their differential diagnosis and consider prescribing antimicrobial therapy.

## Background

In recent years we have become increasingly aware of the diversity of pathogens that ticks may transmit to humans or animals [1–5]. Pathogen transmission typically occurs when humans or other animals come into contact with infected ticks, some of which feed indiscriminately on vertebrate hosts. Consequently, occupations or populations that frequently enter tick habitats are at an increased risk of tick-borne disease. This holds true in Mongolia, where pastoral-herders, who make up 26% of the country’s population of 3 million, spend prolonged periods of each day outdoors moving livestock from pasture to pasture. Despite this, little is known about the scope of tick-borne disease in Mongolia. While epidemiological studies examining spotted fever group (SFG) *Rickettsia* have been conducted [6–8], few have employed molecular techniques. Several additional studies have examined tick-borne encephalitis virus (TBEV), a serious tick disease found in northern Mongolia [9–12]. As a number of rural Mongolians have developed severe illnesses subsequent to tick bites, at the request of the Ministry of Health we conducted a survey for ticks in three ecologically unique regions: forested taiga, central steppe grassland, and the Gobi region of Mongolia. By implementing molecular detection methods, we sought to describe the genetic profiles and distribution of SFG *Rickettsia* among ticks located in varying ecotones and latitudes, across central Mongolia.

## Methods

**Fig. 1:**
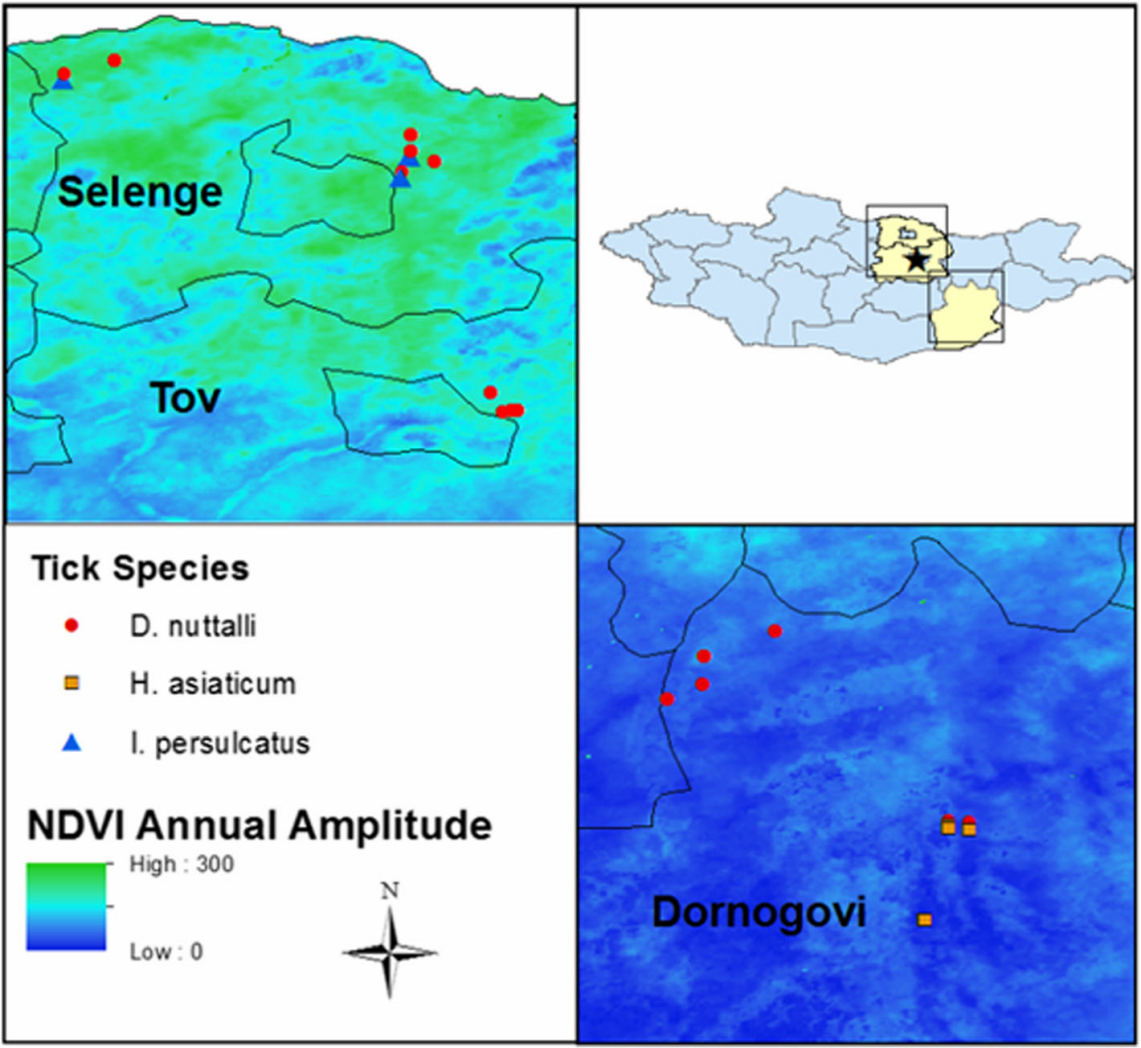
Tick species distribution overlaid on vegetation coverage

A total of 1497 adult unfed ticks: 261 *Ixodes persulcatus*, 795 *Dermacentor nuttalli*, and 441 *Hyalomma asiaticum*, were collected from three provinces; Selenge, Tov and Dornogovi, which stretch vertically across central Mongolia. Nineteen sites were selected for sampling based on their diverse landscape, elevation (range 691–1616 m above sea level), vegetation coverage (Table 1), and proximity to previous sampling locations of ongoing human and livestock tick-borne pathogen surveillance. Basic information pertaining to tick habitat was inferred by using annual amplitude of vegetation coverage (Normalized Difference Vegetation Index) linked to sample GPS points, with tick species and vegetation coverage depicted in Fig. 1. [13].

**Table 1:** Summary of unfed adult ticks and their environment’s characteristics including elevation above sea level and Normalized Difference Vegetation Index (NDVI), collected in Mongolia, from April 24^th^ to May 16^th^ 2015

Aimag	Tick species	Ticks/pools	Mean NDVI annual amplitude (range)	Elevation above sea level (m)
Tov	*Dermacentor nuttalli*	358/71	208.5 (205–249)	1500–1615
Selenge	*D. nuttalli*	169/34	266.6 (242–351)	691–931
	*Ixodes persulcatus*	261/53	261 (255–351)	809–931
Dornogovi	*D. nuttalli*	268/54	89.1 (58–112)	895–1331
	*Hyalomma asiaticum*	441/88	21 (20–61)	757–929

Sampling occurred between April 24^th^ and May 16^th^ 2015, with 884 unfed ticks collected from the environment by dragging, and an additional 613 unfed ticks removed from livestock returning from pasture. All immature life stages of ticks and engorged or partially blood fed adult ticks were excluded from analysis, as some pathogens are killed by host complement system during tick feeding.

After collection, live ticks were stored at -80 °C in vials separated by genus (based on visual identification of morphological characteristics) and sampling location. A total of 299 pools of five ticks each (±1) were aggregated by tick genus and sampling site. Ticks were then homogenized with sterile mortar and pestle, with homogenate centrifuged in PBS to collect supernatant. Genomic DNA and RNA were manually extracted from tick supernatant using TaKaRa kits (TaKaRa Bio Inc, Shiga, Japan), according to the manufacturer’s instructions. Species were then identified using the partial mitochondrial 16S rRNA gene [14] targeting *Ixodes*, *Hyalomma* and *Dermacentor* tick DNA.

A nested PCR assay was used to amplify the outer membrane protein A (*ompA*) and citrate synthase (*gltA*) gene, using the Thermo Scientific DreamTaq Green PCR Master Mix (Thermo Scientific, Waltham, USA). Primer for *ompA* include Rr190.70p/Rr190.602n (530 bp) outer primers and 190.70-38s1/190.602-384r1 (340 bp) inner primers, which have been used in similar studies [15–18]. Primers for *gltA* include CS2d/CSEndr outer primers (1300 bp) and RpCS877p/RpCS1258n (381 bp) inner primers [16]. Amplified product was visualized on a 1.2% (w/v) agarose gel using gel electrophoreses, with positive amplicons directly sequenced on a 3730 Sequencer (Applied Biosystems, Carlsbad, USA) by a commercial company (Tianyi, Beijing, China). The assembled sequences were compared against the NCBI nt/nr database (PubMed website: https://blast.ncbi.nlm.nih.gov/Blast.cgi) and later submitted to GenBank. Phylogenetic analysis was conducted using Molecular Evolutionary Genetics Analysis (MEGA) software, version 5.

RNA from *I. persulcatus* and *D. nuttalli* tick pools collected from Selenge and Tov aimags, were also screened for TBEV on the 7500 Real-Time PCR System (Applied Biosystems, Waltham, USA), using a commercial real-time PCR (qRT-PCR) kit provided by Liferiver™, (Shanghai ZJ Bio-Tech Co., LTD, Shanghai, China), in which TBEV RNA was transcribed into cDNA and then amplified, according to the manufacturer’s instructions. A maximum likelihood estimate (MLE) and a minimum infection rate (MIR) were calculated using PooledinRate software (http://www.cdc.gov/westnile/resourcepages/mosqSurvSoft.html) to predict pool infection rates based on number of ticks per pool and infection status of pool.

## Results and discussion

Overall pool detection prevalence in *I. persulcatus* ticks was 92.5% (49/53) for “*Candidatus* R. tarasevichiae” and 1.9% (1/53) for TBEV (Table 2). *Rickettsia raoultii* was found in 72.8% (115/158) of *D. nuttalli* pools, with Terelj, a scenic area with high rates of international tourism, having 60/71 (84.5%) pools testing positive. All sequences were submitted to GenBank (accession numbers KU361212–KU361217).

**Table 2:** Summary of molecular study results from ticks collected in Mongolia April 24th and May 16th 2015

Tick species	Soum (aimag)	Pathogens detected (%)		
		*R. raoultii*	"*Candidatus* R. tarasevichiae"	TBEV
*D. nuttalli*	Terelj (Tov)	60/71 (84.5)	0	0/46
	Dalanjargalan (Dornogovi)	29/51 (56.8)	0	nd
	Eroo (Selenge)	21/28 (75)	0	0/28
	Sainshand (Dornogovi)	1/2 (50)	0	nd
	Tushig (Selenge)	4/6 (66.6)	0	0/6
	Totals	115/158 (72.8)	0	0/80
*H. asiaticum*	Sainshand (Dornogovi)	0	0	nd
*I. persulcatus*	Eroo (Selenge)	0	48/52 (92.3)	1/52 (1.9)
	Tushig (Selenge)	0	1/1 (100)	0/1
	Totals	0	49/53 (92.5)	1/53 (1.9)
Totals		115/299 (38.5)	49/299 (16.4)	1/133 (0.1)

*Abbreviations*: *nd* not determined

The MLE and MIR of *R. raoultii* in *D. nuttalli* ticks and “*Candidatus* R. tarasevichiae” in *I. persulcatus* ticks can be found in Table 3. Briefly, calculations of MLE for *R. raoultii* in *D. nuttalli* ticks ranged from 15.1 to 30.4% with lower rates observed in the south compared to a MIR ranging from 11.2 to 16.6%. The MLE of “*Candidatus* R. tarasevichiae” in *I. persulcatus* ticks was 46.6% (95% CI: 35.2–63.6%) compared to a MIR of 19.5% (95% CI: 14.7–24.4%). All 88 pools of *H. asiaticum* ticks tested negative for *Rickettsia* DNA. All “*Candidatus* R. tarasevichiae”-infected ticks were collected in Selenge aimag, which is a known habitat for *I. persulcatus* in Mongolia [7, 19, 20].

**Table 3:** Using minimum infection rates and maximum likelihood estimations to calculate tick infection rates by species and aimag

Aimag	Species	Pathogen	Minimum infection rate (95% CI)	Maximum likelihood infection rate (95% CI)
Selenge	*I. persulcatus*	"*Candidatus *R. tarasevichiae"	19.5 (14.7–24.4)	46.6 (35.2–63.6)
Selenge	*D. nuttalli*	*R. raoultii*	14.8 (9.4–20.2)	22.6 (15.5–32.2)
Tov	*D. nuttalli*	*R. raoultii*	16.6 (12.9–20.6)	30.4 (23.8–38.6)
Dornogovi	*D. nuttalli*	*R. raoultii*	11.2 (7.4–15.0)	15.1 (10.6–20.9)

**Fig. 2:**
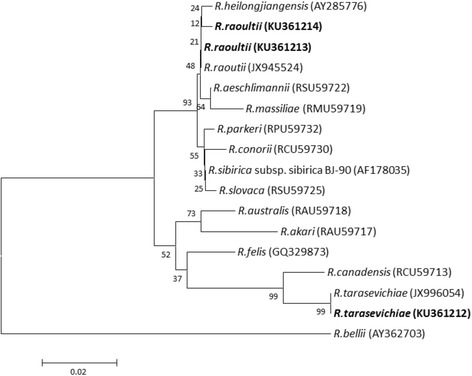
Phylogenetic analysis was conducted based on 341-bp *gltA* gene sequence data with the neighbour-joining method under a Kimura 2-parameter model using MEGA software, version 5.0. Bootstrap analysis of 10,000 replicates was oriented by using *R. bellii* as the outgroup. *Numbers* on the branches indicate percentage of replicates that reproduced the topology for each clade. *Scale-bar* indicates estimated evolutionary distances. The GenBank accession number is listed at the end of each strain. The sequences in *bold* are the ones identified in this study

**Fig. 3:**
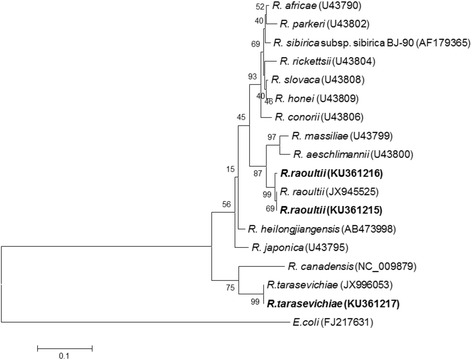
Phylogenetic analysis was conducted based on 322-bp outer membrane protein A (*ompA*) gene sequence data with the neighbour-joining method under a Kimura 2-parameter model using MEGA software, version 5.0. Bootstrap analysis of 10,000 replicates was oriented by using *E. coli* as the outgroup. *Numbers* on the branches indicate percentage of replicates that reproduced the topology for each clade. *Scale-bar* indicates estimated evolutionary distances. The GenBank accession number is listed at the end of each strain. The sequences in *bold* are the ones identified in this study

Based on the phylogenetic analysis of 341 bp *gltA* gene sequence data, we documented two sequences of *R. raoultii* and one “*Candidatus* R. tarasevichiae” sequence. One of the *R. raoultii* sequences (KU361213) shared 100% identify with *R. raoultii* (JX945524) which was detected in two patients in China [21], with the other sequence (KU361214) differing from the Chinese sequence by one base. The “*Candidatus* R. tarasevichiae” of our study (KU361212) shared 100% identity with the sequence JX996054 recovered from human cases of “*Candidatus* R. tarasevichiae” in China [15]. Phylogenetic analysis based on 322 bp *ompA* gene sequence data determined one of two *R. raoultii* sequences (KU361215) and the only “*Candidatus* R. tarasevichiae” sequence (KU361217) recovered in our study shared 100% identity with sequences JX945525 and JX996053 from human cases in China [15, 22]. The other *R. raoultii* sequence showed 99.4% similarity to that of the human patient in China with 2 bp difference. Phylogenetic trees depicting variation by species and gene fragment can be found in Figs. 2 and 3.

Previous entomological surveys focusing on ticks in Mongolia have reported a countrywide distribution of *D. nuttalli*, while *H. asiaticum* and *I. persulcatus* have clearly defined ecological niches on opposite sides of Mongolia [23], thus influencing the geographical distribution of species-specific tick-borne diseases. Living in close proximity to coniferous regions in the northern aimags of Mongolia likely remains a significant risk factor for exposure to diseases transmitted by *I. persulcatus* hard ticks [7, 11, 12, 19, 20, 24–26]. *Rickettsia raoultii* detection among *D. nuttalli* ticks were observed in all five sampling districts, suggesting a wider distribution of disease, even extending into China as indicated by other reports [21, 27]. These findings expand upon previous findings which described *R. raoultii* as the predominant SFG *Rickettsia* infection found in 179 *D. nuttalli* ticks collected in four northern Mongolian provinces [7].

## Conclusion

The presence of “*Candidatus* R. tarasevichiae” in ticks should be of concern, given that the *ompA* and *gltA* sequences from this study shared 100% identity with severely ill cases of “*Candidatus* R. tarasevichiae” infection that were recently described [15, 22]. To date there has only been one other study that has reported evidence of “*Candidatus* R. tarasevichiae” in Mongolia [8]. While little is known about the full clinical spectrum of this disease, some patients in China infected with “*Candidatus* R. tarasevichiae” presented with meningitis-like symptoms that are atypical to SFG rickettsiosis, resulting in initial misdiagnosis. This may prove problematic in Mongolia, where TBEV infections commonly presents with fever and meningitis-like symptoms, and is considered a high risk disease in Selenge. However, our analysis of ticks in this region indicated only one positive *I. persulcatus* pool for TBEV (1.9%), which matches similar reports of TBEV from other studies in this region [11, 19]. This is in contrast to the 92.5% positive pool detection prevalence or 46.6% tick infection rate based on MLE, of “*Candidatus* R. tarasevichiae” from the same tick pools.

Future studies are needed to determine if “*Candidatus* R. tarasevichiae” or *R. raoultii* are causing clinical disease in Mongolia. Until then, both Mongolian nationals and foreign travellers who visit the countryside during late spring and summer, should take additional precautions to prevent exposure to ticks and be aware of the risks they pose. Mongolian physicians treating suspected cases of TBEV should include “*Candidatus* R. tarasevichiae” infection in their differential diagnosis and consider prescribing antimicrobial therapy.
